# Expression profiles for six zebrafish genes during gonadal sex differentiation

**DOI:** 10.1186/1477-7827-6-25

**Published:** 2008-06-30

**Authors:** Anne Jørgensen, Jane E Morthorst, Ole Andersen, Lene J Rasmussen, Poul Bjerregaard

**Affiliations:** 1 Department of Science, Systems and Models, Roskilde University, Universitetsvej 1, DK-4000 Roskilde, Denmark; 2 Institute of Biology, University of Southern Denmark, Campusvej 55, DK-5230 Odense M, Denmark

## Abstract

**Background:**

The mechanism of sex determination in zebrafish is largely unknown and neither sex chromosomes nor a sex-determining gene have been identified. This indicates that sex determination in zebrafish is mediated by genetic signals from autosomal genes. The aim of this study was to determine the precise timing of expression of six genes previously suggested to be associated with sex differentiation in zebrafish. The current study investigates the expression of all six genes in the same individual fish with extensive sampling dates during sex determination and -differentiation.

**Results:**

In the present study, we have used quantitative real-time PCR to investigate the expression of ar, sox9a, dmrt1, fig alpha, cyp19a1a and cyp19a1b during the expected sex determination and gonadal sex differentiation period. The expression of the genes expected to be high in males (ar, sox9a and dmrt1a) and high in females (fig alpha and cyp19a1a) was segregated in two groups with more than 10 times difference in expression levels. All of the investigated genes showed peaks in expression levels during the time of sex determination and gonadal sex differentiation. Expression of all genes was investigated on cDNA from the same fish allowing comparison of the high and low expressers of genes that are expected to be highest expressed in either males or females. There were 78% high or low expressers of all three "male" genes (ar, sox9a and dmrt1) in the investigated period and 81% were high or low expressers of both "female" genes (fig alpha and cyp19a1a). When comparing all five genes with expected sex related expression 56% show expression expected for either male or female. Furthermore, the expression of all genes was investigated in different tissue of adult male and female zebrafish.

**Conclusion:**

In zebrafish, the first significant peak in gene expression during the investigated period (2–40 dph) was dmrt1 at 10 dph which indicates involvement of this gene in the early gonadal sex differentiation of males.

## Background

The mechanism underlying sex determination in zebrafish is largely unknown. Currently, no morphological differences in the chromosomes of the two sexes have been identified neither by classical karyotyping nor from the zebrafish genome sequencing project. This indicates that sex determination in zebrafish is mediated by genetic signals from autosomal genes and not by XY/XX or ZZ/ZW sex determination [1-3]. Furthermore, the mammalian sex determining factor *Sry *(located on the Y chromosome) and the sex determining gene *dmy *(*dmrt1y*) located on the Y chromosome in Japanese medaka *Oryzias latipes *[4,5] does not appear to be present in the zebrafish genome. Current knowledge on sex determination and differentiation in zebrafish includes that zebrafish are sexually mature after three months, and that separate sexes can be detected by gonad histology at approximately 40 days post hatch (dph). Furthermore, all zebrafish develop ovary-like gonads regardless of genetic background prior to sex differentiation [3]. Ovarian development is initiated at approximately 10 dph and progresses until 20 dph. At 20 dph until approximately 30 dph testis development is initiated in males simultaneously with ovarian cell apoptosis [2,3].

A number of genes (*dmrt1*, *sox9a*, *amh*, *wt1*, *ftz-f1, gata*) have previously been associated with the process of sex determination or differentiation in zebrafish [2]. None of these genes are suggested to be the single factor responsible for specifying sex in zebrafish [2]. However, the expression pattern and function of these genes suggest that they are part of a signalling network responsible for the development of sex specific gonads [2]. Current research in medaka *Oryzias latipes *suggests a potential signalling pathway in XY individuals of: *dmy*, *sox9a *and *dmrt1 *with *dmy *blocking meiosis, *sox9a *regulating testicular tubule development and *dmrt1 *being important in spermatogenesis [6]. Also, the research in medaka *Oryzias latipes *suggests an important role of *fig α *as well as *cyp19a1a *and *cyp19a1b *in sexual development of gonads in XX individuals [7].

The *Dmrt1 *gene belongs to a group of multiple genes containing a zinc-finger-like DNA-binding motif (DM domain) that has been identified in both invertebrates and vertebrates [8]. Interestingly, the medaka sex determining gene *DMY *(*dmrt1y*) originates from a *dmrt1 *duplication, and sex determining homologues have been identified in the fruit fly *Drosophila melanogaster *(*doublesex *gene) and the worm *Caenorhabditis elegans *(*mab-3 *gene) [8]. In zebrafish, *in situ *hybridization to gonads showed that *dmrt1 *was expressed in developing germ cells of both testis and ovary, suggesting that the *dmrt1 *gene is not only associated with testis development, but may also be important in ovary differentiation of zebrafish [8]. In diverse species including frog, turtle, alligator, bird, and mouse, the *dmrt1 *gene is expressed at higher levels in males compared to females, suggesting that high expression is necessary for testicular differentiation, whereas lower expression is compatible with ovarian differentiation [9-12]. The *Sox *(*SRY*-related genes containing a HMG box) gene family encodes an important group of developmental regulators involved in sex determination. The HMG (high mobility group) box that characterises Sox proteins is a DNA-binding domain and proteins encoded by *Sox *genes act as transcription factors. *Sry*, the founder member of the *Sox *gene family, is the Y chromosomal male determinant in most mammals [13-15]*Sry *is a poorly conserved gene that appears to be exclusive to mammals. In contrast, *Sox*9 is a conserved gene present in all vertebrate types. Like *Sry*, *Sox*9 is required for testis development in mammals, and *Sox9 *deficiency can result in sex reversal in human males. The expression of *Sox9 *during gonadal differentiation is up-regulated in testis and down-regulated in ovaries in mammals, birds and turtles [16]. However, the organisation and function of the *Sox *gene family is less understood in other types of vertebrates and despite the wide distribution of *Sox9 *genes in fish, only few have been investigated [16,17]. In zebrafish, two *sox9 *genes are present *sox9a *and *sox9b*) and their expression patterns indicate that they have unique functions during development [16]. In adult zebrafish the *sox9a *transcript was observed in testis but not in ovary. Conversely, *sox9b *transcripts were detected in ovary, but not in testis [16].

Androgens play a key role in male sex differentiation and sex maturation in vertebrates, including teleosts [18]. The most important androgens in teleost fish are 11-ketotestosterone and testosterone and their action is mediated through specific nuclear androgen receptors (*ar*) [19-21]. In sea bass (*Dicentrarchus labrax*) expression of the *ar *was measured in the gonads during development of a male-dominated population and a female-dominated population [22]. The expression patterns of the two populations were different, with a peak in the *ar *expression in the male-dominated population coinciding with the time of sex differentiation in sea bass [22]. We recently identified and conducted the initial characterisation of a novel androgen receptor from zebrafish [23] and in another recent study, expression of this receptor was higher in the transforming testis compared to ovary suggesting a role during male gonadal differentiation [24]. A key enzyme balancing the ratio of steroid hormones is cyp19 (aromatase) which is the terminal enzyme in the steroidogenic pathway. It converts androgens (e.g., testosterone) into estrogens (e.g., estradiol). Regulation of this gene dictates the ratio of androgens to estrogens; therefore, appropriate expression of this enzyme is critical for sex differentiation and reproduction in vertebrates. Most vertebrates have a single *CYP19 *gene that is regulated by multiple tissue-specific promoter regions. However, the zebrafish has two genes (*cyp19a1a *and *cyp19a1b*) encoding different proteins and each possessing its own regulatory mechanism [1]. In general, the gonadal form *cyp19a1a *is more abundant than the brain form (*cyp19a1b*). The expression of the two *cyp19 *genes has previously been investigated from 0–41 days post fertilisation (dpf) which is the expected time of sex determination and differentiation in zebrafish. *cyp19a1a *expression was highest shortly after hatch from 4–8 dpf. The pattern of *cyp19a1b *expression was segregated into two populations, suggesting an association with sex differentiation [1]. *fig α *is a germ cell-specific transcription factor required for ovarian follicle formation. *fig α *is involved in the coordinate expression of the zona pellucida (zpc) genes. The expression of *fig α *and *zpc *coincides with the onset of gonadal differentiation in zebrafish at 22 dpf [25]. Previous in situ hybridisation studies of *fig α *expression in zebrafish have shown that *fig α *is expressed abundantly in ovaries of adult fish whereas no *fig α *signal could be detected in adult zebrafish testes [25]. Furthermore, at 30 dpf the expression of *fig α *and *zpc *was investigated in eight fish. In five fish both genes were expressed whereas no expression was detected in the last three fish. This indicates female restricted expression of *fig α *in zebrafish [25].

Since previous studies indicate involvement of *ar*, *sox9a, dmrt1, cyp19a1b, cyp19a1a *and *fig α *in sex differentiation in vertebrates, including fish, we found it interesting to investigate the expression of these six genes during sex determination and differentiation in zebrafish. The idea is to measure the expression of all genes in the same individual, thereby allowing comparison of expression. This might lead to identification of putative male and female zebrafish and indicate whether one or more of these genes could function as an early genetic sex marker in zebrafish. Therefore, the aim of the current study is to determine the expression of the *ar*, *sox9a, dmrt1, cyp19a1b, cyp19a1a *and *fig α *during the developmental period in which sex determination and gonadal differentiation takes place in zebrafish.

## Methods

### Animals

Juvenile zebrafish originated from a brood population of fish. In the evening breeding boxes were placed in an aquarium with parent fish and eggs were collected the following morning. The eggs were sorted into fertilised and non-fertilised and the fertilised eggs were placed in 900 ml glass beakers and kept at 26 +/- 1°C and a light-dark period of 14:10 h. In the interval 3–22 dph the larvae were fed two times daily with powdered dry food (Sera Micron) and one time daily with newly hatched artemia sp. nauplii (Intér Ryba GmbH, Germany). For a period of three days (23–25 dph) the powdered dry food was given in combination with TetraMin Baby Powder Food. From 26 dph and onwards the dry food consisted solely of TetraMin Baby Powder Food. Artemia was still given once daily. At 2, 4, 6, 8, 10, 12, 14, 16, 18, 19, 20, 21, 22, 25, 30 and 40 dph zebrafish were frozen individually in liquid nitrogen and stored at -80°C until further analysis.

### RNA purification

Total RNA was purified from whole fish for the AR expression during sex determination and differentiation experiment. Zebrafish or zebrafish tissue was homogenised for 20 sec. using an Ultra-Turrax homogenizer (IKA-Werke). For purification of RNA, Total RNA Isolation Kit (Macherey-Nagel) was used according to manufacturer's instructions. RNA concentration was measured spectrophotometrically (Gene-Quant, Pharmacia Biotech), checked by gel electrophoresis (1.2% agarose gel) and stored at -80°C until further use. cDNA was obtained from 0.3 μg RNA using SuperScript II reverse transcriptase kit with Oligo dT primer (Invitrogen) according to manufacturers instructions.

### Quantitative RT-PCR

Expression of *Danio rerio ar*, *sox9a*, *dmrt1*, *cyp19a1b*, *fig α *and *cyp19a1a *was analysed by qRT-PCR using a real-time light-cycler (Roche). As zebrafish is an established model-organism, the genome has been sequenced and the seventh assembly is available. All genes investigated have previously been identified and sequences were available in GenBank. Primers for qRT-PCR analysis were designed using the Primer3 program [26] (Table 1). The final PCR reactions contained: 0.4 mM of each primer; 0.25 × SYBR Green (Invitrogen); 4 mM MgCl_2 _and as template 5 μl of cDNA reverse transcribed from a standardized amount of total RNA (0.3 μg). qRT-PCR was performed using Hotstart Taq polymerase (Qiagen) in a final volume of 20 μl. All quantitative reactions were subjected to: 95°C for 15 min followed by 45 cycles at 94°C for 15 s, 59°C 15 s and 72°C 15 s. Melting curve analysis was applied to all reactions to ensure homogeneity of the reaction product. In addition, the amplicon size was checked electrophoretically for each primer set and subsequent sequencing revealed that it corresponded to the zebrafish *ar*, *sox9a*, *dmrt1*, *cyp19a1b, fig α *and *cyp19a1a*, thus verifying the identity of the genes. Potential contamination was assessed by including non-reverse transcribed total RNA (genomic DNA contamination) and no-template controls. No products were observed in these reactions. Dilution curves generated by serial dilutions (1:10) of cDNA were used to calculate amplification efficiencies according to the Roche protocol. All assays were quantitative with standard curve (mean threshold cycle [C_t_] vs. log cDNA dilution) slopes of -3.96 (*β-actin*), -2.10 (*sox9a*), -3.39 (*dmrt1*), -2.88 (*ar*), -3.00 (*cyp19a1a*), -4.22 (*cyp19a1b*) and -3.68 (*fig α*), translating to relatively high PCR efficiencies (E) of 1.79 (*β-actin*), 3.01 (*sox9a*), 1.97 (*dmrt1*), 2.23 (*ar*), 2.15 (*cyp19a1a*), 1.73 (*cyp19a1b*) and 1.87 (*fig α*). over the detection range, the linear correlation (R_2_) between the mean C_t _and the log cDNA dilution was > 0.99 in each case. Transcript levels of the target genes were normalized to *D. rerio *β-actin after correcting for differences in amplification efficiencies.

**Table 1:** Oligonucleotide primers used for quantitative real time PCR analysis.

Primer	Amplicon	Sense primer 5'-3'	Antisense primer 5'-3'
ar	242 nt	AGCAGCAGCACCACTACCA	TTCCTTCCTGCCTCTCGTTC
sox9a	719 nt	CGGTGAAGAACGGCCAGAGC	CTGTAGAGTCAGCAATGGGT
cyp19a1b	230 nt	AACATTGGACGCATGCATAA	TGTTTGATGGTGCTGATGGT
dmrt1a	151 nt	ATGGCAGAGCAGAACGATTT	TAGTCCCACAACAGCATGGA
fig α	663 nt	ATGTCGTGTGAAATGACCGGC	CTAGGATGGGAGTGAACTTGG
cyp19a1a	131 nt	AGATGTCGAGTTAAAGATCCTGCA	CGACCGGGTGAAAACGTAGA
β-actin	272 nt	CCTGACCGAGAGAGGCTACA	CGCAAGATTCCATACCCAAG

### Tissue expression

Adult zebrafish, 5 males and 5 females were anaesthetized in a buffered solution of MS-222 (0.1 g/l) and quickly dissected into brain, gonads, liver, eyes, spleen, heart, gut, gall bladder, muscle and gills. The dissected tissues were immediately frozen in liquid nitrogen and stored at -80°C. The RNA purification and quantitative qRT-PCR was conducted as described for the gene expression during sex determination and differentiation experiment except that approximately 5 mg of zebrafish tissue were used for total RNA purification. Also, 1 μg total RNA was reverse transcribed using SuperScript II reverse transcriptase kit with Oligo dT primer (Invitrogen) according to manufacturers instructions. Following the qRT-PCR, the reactions were spinned down and loaded on a 0.8% agarose gel.

### Data handling and statistical analysis

To analyse the expression patterns of *ar*, *sox9a*, *dmrt1, fig α, cyp19a1a *and *cyp19a1b *during the investigated period, it was necessary to discriminate between individual fish with high and low gene expression. This was done using the GraphPad Prism program makes a cut-off value between the high and low expressers as indicated on the graph, which is generated automatically in the program when making a two-segment graph. The investigated fish were a mixture of males and females and since large differences in expression were observed during the investigated period, it was difficult to separate the different effects from each other without this distinction between high and low expressers.

As expression of all the six investigated genes has been measured on individual fish, it is possible to compare the expression patterns. First, *ar*, *sox9a *and *dmrt1 *genes with expected high expression in males were divided into high and low expressers, then for each individual fish the expression levels (high or low) of the three genes were compared. Next, the percentage of individuals with high or low expression of the three genes was calculated. Secondly, the same was calculated for the two genes (*fig α *and *cyp19a1a*) with expected high expression in females. Third, the percentage of individuals with expression patterns as expected for male or female for all five genes was calculated.

Gene expressions in zebrafish during the sex determination and differentiation period were statistically analysed for variance. Data were normally distributed with similar variance after log transformation and a two-way ANOVA (variables: high/low expressers and dph) analysis was followed by Tukey test with a significance level of (p < 0.05).

## Results

**Figure 1:**
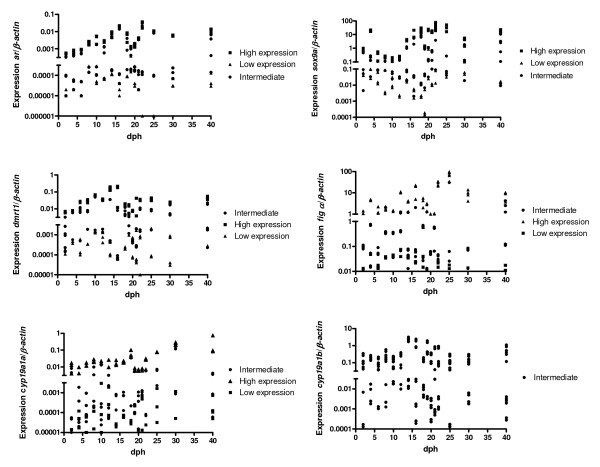
**Expression of *sox9a*, *dmrt1*, *ar*, *cyp19a1b*, *fig α *and *cyp19a1a *in individual juvenile zebrafish during the investigated period from 2–40 dph.** On each sampling date 12 individuals were investigated. The cut-off value between high and low expressers is indicated on each of the figures by the segmented Y-axis. The expression pattern of *ar*, *sox9a*, *dmrt1*, *fig α *and *cyp19a1a *were compared in each individual zebrafish, three genes with expected high expression in males (*ar*, *sox9a*, *dmrt1*) and two genes with expected high expression in females (*fig α*, *cyp19a1a*). Individuals with expression pattern of all five genes that were as expected for either male or female corresponded to 56% and symbols ■ were used for the tentative identification of males and ▲ were used for the tentative identification of females. Individuals in which the expression of all five genes was not as expected for either female or male corresponded to 44% and were called intermediate ●. *cyp19a1b *was not included in the tentative identification of sex (see text for explanation) and therefore the ● symbol was used.

To analyse the expression patterns of *ar*, *sox9a*, *dmrt1a, fig α, cyp19a1a *and *cyp19a1b *during the expected sex determination and differentiation period, individuals were divided into groups of high and low expressers based on the method described previously where the distribution between investigated individuals and the cut-off value between high and low expressers are shown in Figure 1 for each of the investigated genes. The expression pattern of *ar*, *sox9a*, *dmrt1*, *fig α *and *cyp19a1a *were compared in each individual zebrafish, three genes with expected high expression in males (*ar*, *sox9a*, *dmrt1*) and two genes with expected high expression in females (*fig α*, *cyp19a1a*). The data show that for *ar*, *sox9a *and *dmrt1 *expression 78% were either high or low expressers of all three genes. For *fig α *and *cyp19a1a *expression, 81% were either high or low expressers of both genes. Individuals with expression pattern of all five genes that were as expected for either male or female corresponded to 56% and square symbols were used for the tentative identification of males and triangles were used for the tentative identification of females. Individuals in which the expression of all five genes was not as expected for either female or male corresponded to 44% and were called intermediate and a round symbol was used. *cyp19a1b *was not included in the tentative identification of sex and therefore the round symbol was used. There was always more than 10 times difference in expression between the two groups and statistical analysis showed that for all investigated genes there was a significant difference (p < 0.05) between high and low expresser groups. The ratio between genes expected to be primarily "female" and genes expected to be primarily "male" were calculated (Table 2) together with ratios for *fig α*/*dmrt1*.

**Table 2:** Ratio between genes with expected sex-related expression

	Ratio	Average	± Std
Expected females		118.51	98.01
Expected males	$\frac{\left(fig\alpha \times cyp19a1a\right)}{\left(dmrt1\times sox9a\times ar\right)}$	1.12 × 10^-7^	2.08 × 10^-7^
Female gonad		8.54 × 10^16^	3.65 × 10^15^
Male gonad		7.49 × 10^-16^	5.13 × 10^-17^

Expected females		2.18	3.70
Expected males	$\frac{fig\alpha }{dmrt1}$	0.002	0.003
Female gonad		11995.65	3061.41
Male gonad		0.017	0.0044

The calculated ratios of expected females and expected males are based only on data from the 56% of the investigated fish that showed expected "male" or "female" expression pattern for all five genes (*fig α*, *cyp19a1a*, *dmrt1a*, *sox9a *and *ar*). The ratios of female gonad and male gonad are based on adult fish.

### *ar *expression

**Figure 2:**
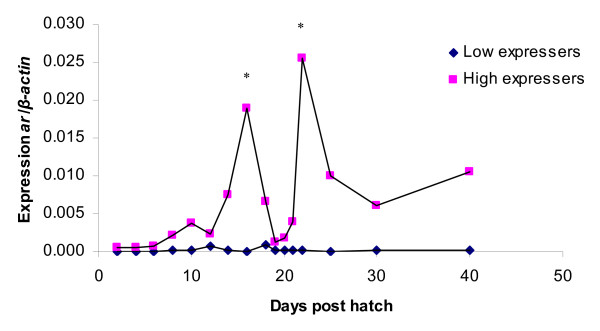
**Expression of androgen receptor (*ar*) in whole juvenile zebrafish homogenate during sex determination and differentiation.** Values are expressed as arbitrary units of *ar *expression levels normalised against the expression levels of *β-actin *amplified from the same template. * indicate significant difference (p < 0.05) after Tukey test compared to the day before in the high expresser group. There was always significant difference between the high and low expresser group (p < 0.05) after Tukey test.

The expression levels of *ar *were low for both the high and low expresser group during the first period (2–8 dph) (Figure 2), however, with two distinct groups of high and low expressers. The *ar *expression has a peak at 16 dph, which is significantly different (p < 0.05) compared to the expression on the day before. The *ar *expression reaches the highest level at 22 dph for the high-expresser group, also significantly different (p < 0.05) from the expression on the day before. The 22 dph peak corresponds to the time period right after the bipotential gonad is starting to differentiate into ovary or testes. In the low expresser group no increase in *ar *levels was seen. In the period 19–20 dph there is a marked decrease in the expression level for the high-expresser group, which coincides with the time of juvenile ovary-to-testes transformation in zebrafish.

### *sox9a *expression

**Figure 3:**
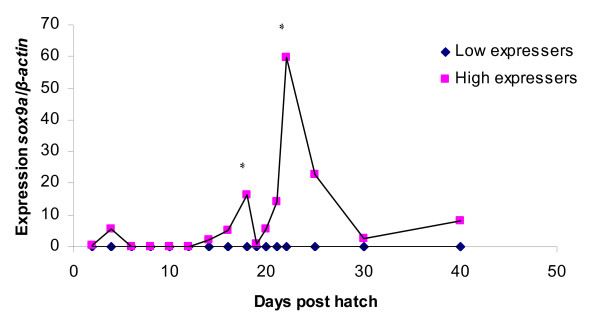
**Expression of *sox9a *in whole juvenile zebrafish homogenate during sex determination and differentiation. **Values are expressed as arbitrary units of *sox9a *levels normalised against the levels of β-actin amplified from the same template. * indicate significant difference (p < 0.05) after Tukey test compared to the day before in the high expresser group. There was always significant difference between the high and low expresser group (p < 0.05) after Tukey test.

The expression of *sox9a *is very low for the low expresser group during the entire investigated period. In the high expresser group there are three peaks, at 4, 18 and 22 dph (Figure 3). The peak in the high expresser group at 4 dph is very early in development and the expression levels at 2 dph and 4 dph are not significantly different (p > 0.05). Interestingly, the peak at 18 dph which is significantly different compared to the expression of *sox9a *at 16 dph is just before the time of oocyte apoptosis (20–23 dph) in individuals expected to develop into males and might therefore represent a signal in the male developmental pathway. The peak at 18 dph is followed by a rapid decrease and very low *sox9a *expression at 19 dph. At 22 dph the expression level in the high-expresser group peaks again with expression level significantly different (p < 0.05) from that at 21 dph. The 22 dph peak in *sox9a *expression coincide with an expression peak in *ar *in the high expresser group.

### *dmrt1 *expression

**Figure 4:**
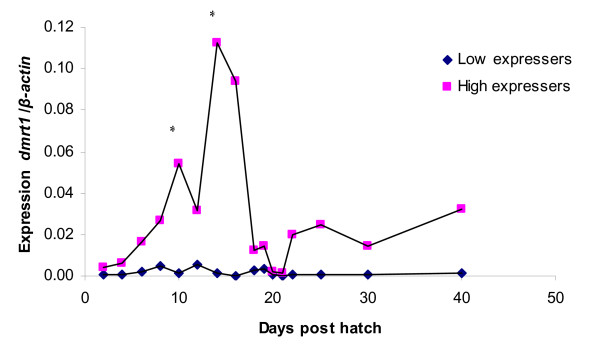
**Expression of *dmrt1 *in whole juvenile zebrafish homogenate during sex determination and differentiation. **Values are expressed as arbitrary units of *dmrt1 *levels normalised against the levels of β-actin amplified from the same template. * indicate significant difference (p < 0.05) after Tukey test compared to the day before in the high expresser group. There was always significant difference between the high and low expresser group (p < 0.05) after Tukey test.

The high-expresser group of *dmrt1 *has two distinct expression peaks (at 10 and 14 dph) during the investigated period (Figure 4). Both peaks are significantly different (p < 0.05) compared to the expression during previous days in the high expresser group. The peak in *dmrt1 *expression at 10 dph is early in development and might represent an early marker of male development. The peak at 14 dph is also quite early in development and these results could indicate that *dmrt1 *expression is important in juvenile sex determination and/or differentiation. The 14 dph peak is followed by several days (21–22 dph) with low *dmrt1 *expression which is coinciding with low expression of *ar *and *sox9a *in the high expresser group. The low-expresser group shows a relatively low expression of *dmrt1 *during the entire period, but there might be an indication that at 10 and 16 dph the *dmrt1 *expression decreases in the low-expresser group which is opposite to the expression in the high-expresser group.

### *cyp19a1b *expression

**Figure 5:**
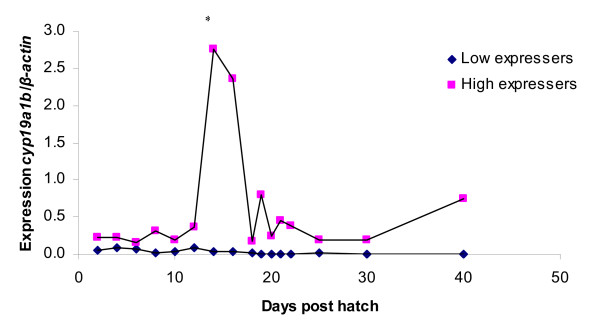
**Expression of *cyp19a1b *in whole juvenile zebrafish homogenate during sex determination and differentiation. **Values are expressed as arbitrary units of *cyp19a1b *levels normalised against the levels of β-actin amplified from the same template. * indicate significant difference (p < 0.05) after Tukey test compared to the day before in the high expresser group. There was always significant difference between the high and low expresser group (p < 0.05) after Tukey test.

In the group of high-expressers of *cyp19a1b*, one distinct expression peak is seen at 14 dph, the level being significantly different (p < 0.05) compared to expression at 12 dph (Figure 5). During the period 18–30 dph small oscillations in expression are seen in the high-expresser group and from 30–40 dph the *cyp19a1b *expression increases. The levels of *cyp19a1b *expression in the low-expresser group are decreasing in the period 6–21 dph. Thereafter the expression level remains very low.

### *fig α *expression

**Figure 6:**
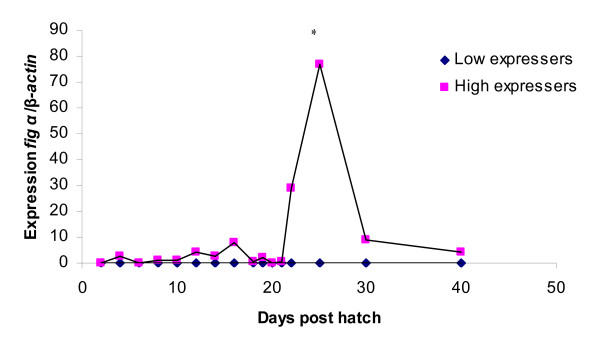
**Expression of *fig α *in whole juvenile zebrafish homogenate during sex determination and differentiation.** Values are expressed as arbitrary units of *fig α *levels normalised against the levels of β-actin amplified from the same template. * indicate significant difference (p < 0.05) after Tukey test compared to the day before in the high expresser group. There was always significant difference between the high and low expresser group (p < 0.05) after Tukey test.

The expression of *fig α *is low with small oscillations in the high expresser groups until 22 dph and the expression in the low expresser groups is almost undetectable during the entire experimental period. A very distinct peak in *fig α *expression in the high expresser group is seen at 25 dph, significantly different (p < 0.05) compared to the expression at 22 dph (Figure 6). The peak in *fig α *expression coincides with the expected onset of gonadal differentiation to ovary in female zebrafish.

### *cyp19a1a *expression

**Figure 7:**
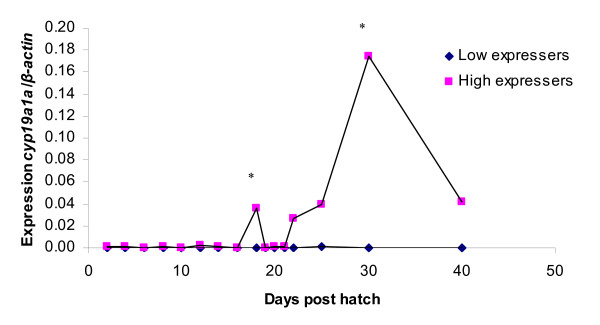
**Expression of *cyp19a1a *in whole juvenile zebrafish homogenate during sex determination and differentiation.** Values are expressed as arbitrary units of *cyp19a1a *levels normalised against the levels of β-actin amplified from the same template. * indicate significant difference (p < 0.05) after Tukey test compared to the day before in the high expresser group. There was always significant difference between the high and low expresser group (p < 0.05) after Tukey test.

The *cyp19a1a *expression in the low expressers is low during the entire investigated period whereas two peaks in expression are seen in the high-expresser groups, at 18 dph and 30 dph, respectively (Figure 7). For both peaks the expression is significantly different (p < 0.05) compared to the expression on the previous day. The peak at 18 dph is just prior to the initiation of gonadal differentiation in zebrafish and the peak at 30 dph is just after the expected time of gonadal differentiation and might reflect an increased estrogen demand in the gonads.

### Tissue expression in adult zebrafish

**Figure 8:**
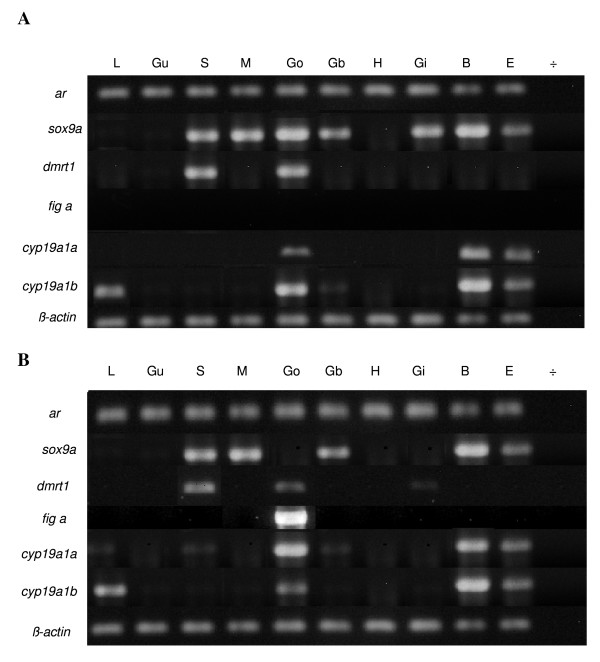
**Tissue specific expression of *ar*, *sox9a*, *dmrt1*, *fig α*, *cyp19a1a *and *cyp19a1b *in adult zebrafish.A **Tissue from male zebrafish. **B **Tissue from female zebrafish. L: liver, Gu: gut, S: spleen, M: muscle, Go: gonad, Gb: gall bladder, H: heart, Gi: gills, B: brain, E: eye, ÷: no DNA (water control).

The tissue specific expression of *ar*, *sox9a dmrt1*, *fig α*, *cyp19a1a *and *cyp19a1b *in adult male and female zebrafish was investigated by qRT-PCR to determine whether the genes were uniformly expressed or restricted to specific tissues or sex. Investigated tissues included liver, heart, brain, spleen, muscle, gall bladder, gut, eyes, gill and gonad (Figure 8). *ar *was expressed in all of the ten different tissues investigated in males and females. The *sox9a *expression was similar in the two sexes except in gonads and gill where it was low in female fish compared to the expression in male fish indicating that this "male" gene still has a role in the adult phase. *dmrt1 *was expressed in spleen and gonads in both sexes, but at highest level in males. The only tissue with expression of the "female" gene *fig α *was female gonads. *cyp19a1a *and *cyp19a1b *were differentially expressed in different organs of male and female zebrafish, liver expression of *cyp19a1a *being higher in male than in female, and higher in female gonads than in male gonads. *cyp19a1b *expression was relatively higher in female than in male brain.

## Discussion

Testicular type *sox9 *is the most upstream conserved gene in the sex determining cascade among vertebrates [29]. Therefore, the expression pattern of this gene during zebrafish sex determination and differentiation is important. In non-mammalian vertebrates like birds and turtles, *sox9 *is expressed in testes and down-regulated in ovaries during gonadal differentiation similar to the expression pattern seen in mammals [30,31]. In the present study, expression of *sox9a *is found in several tissues including spleen, muscle, gall bladder, brain and eye of both male and female zebrafish. However, *sox9a *is also expressed in gonad and gill in a male-specific manner. This is in accordance with a previous study in adult zebrafish showing different expression patterns for *sox9a *and *sox9b *in zebrafish gonads suggesting that *sox9a *retained its function in the testis while *sox9b *possibly acquired a different function in zebrafish ovary during evolution [16]. The expression pattern of *sox9a *in zebrafish during sex determination and differentiation segregated the fish into two groups. In fish of the high expresser group three peaks were observed. The first small peak seen at 4 dph is early in development and might be related to skeletal development, as *sox9 *in mammals is also involved in this process [16], but it could also be related to sex determination and differentiation. The peaks at 18 and 22 dph coincide with the expected time of gonadal differentiation, i.e. ovary to testis transformation in genetic males, which is a key event in zebrafish sex differentiation. In the mammalian bipotential gonad, *Sry *initiates *sox9 *expression and translocation from cytoplasm to nucleus which induces expression of *amh *that is an important testis determining factor [32]. The expression pattern of *sox9a *seen in this study could indicate involvement of *sox9a *in sex differentiation in zebrafish. However, no available data including those of the present study firmly indicates that *sox9a *is the sex determining gene in zebrafish, but merely suggests that it might be involved in the sex signalling cascade and gonadal sex differentiation. This is in agreement with a study in medaka where the level of testicular type *sox9 *(*sox9a2*) expression in somatic cells is equally high in both sexes at the time when *dmy *expression is initiated during early gonadal differentiation. However, during the period from 10–30 dph, *sox9 *expression continues only in the Sertoli cells in male gonads, with a marked reduction in the XX gonads [29].

In a study investigating *amh*, *sox9a*, *sox9b *and *cyp19a1a *expression in undifferentiated gonads of zebrafish, expression of all genes could be detected [33], however, in the differentiated gonads a sexually dimorphic expression pattern was found; *sox9a *and *amh *were expressed in testis whereas *cyp19a1a *was not. In ovaries, *sox9b *and *cyp19a1a *were expressed while *amh *was not [33]. Based on the expression pattern of these genes during sex differentiation, the authors suggested that 17 dph represents a transitional stage in zebrafish gonad development and by 31 dph gonads have differentiated into testes or ovaries [33]. This is in accordance with the present study in which distinct peaks in both *cyp19a1a *and *sox9a *expression was found at 18 dph. Furthermore, these results are in agreement with those of a recent study of the molecular mechanism of the ovary-to-testes transition which indicated that all males go through the juvenile ovary phase until approximately 21 dpf (corresponding to 19 dph). However, they differ in the extent of commitment toward femaleness during this period and can be divided into three types of males based on the intensity, onset and duration of gonadal transformation [3].

In a number of species including humans, mice, chickens, alligators and turtles, the *dmrt1 *expression is limited to the gonads, and the expression is considerably up-regulated in the developing testes compared to ovaries. The timing of this up-regulation varies between species, but generally occurs in the late sex determining or early testis-differentiation period. This characteristic *dmrt1 *expression pattern seen in different species indicates that this gene is specifically involved in the early formation of testes [34]. The results from the present study correspond well with this general notion. We found peaks in expression of *dmrt1 *in the high expresser group prior to the expected time of ovary to testis transformation. The expression peaks of *dmrt1 *early in the sex determination and differentiation period could indicate that it is involved upstream in the signalling cascade that initiates sex determination and differentiation in zebrafish. In a recent model of medaka gonadal development it is shown that the *dmrt1 *expression is detected from 10 dph and is thereby the first gene to be differentially expressed between males and females except for *DMY *[35]. This is in agreement with the results from the present study where we see a peak in *Dmrt1a *expression at 10 dph. However, the idea that the *dmrt1 *gene might be the sex-determining gene in non-mammalian vertebrates has been rejected based on the lack of *DMY *(*Dmrt1bY*) in other fish species than *Oryzias latipes*, including *Oryzias celebensis*, *Oryzias mekongenesis*, guppy *Poecilia reticulata*, pufferfish *Takifugu rubripes *and zebrafish *Danio rerio *[34,36]. The *dmrt1 *gene most likely has a role downstream the sex determination event and it might be involved in testis development in teleost fish analogous to its putative role in mammalian species [34]. This is in accordance with results from medaka where results indicated that *dmrt1 *regulates spermatogonial differentiation [37]. Sexual development in sex-reversed medaka gonads indicates that *DMY *is not necessary for gonadal differentiation and that expression of *fig *α and *dmrt1 *correlate with the phenotypic differentiation of the gonads [7]. The expression pattern of *fig α *in the present study resembles to some extent that in the hermaphrodite fish *Kryptolebias marmoratus*, where *fig α *expression was very low until 39 dpf where expression peaks and remains high until 103 dpf which was the last day measured [38]. Furthermore, *fig α *showed no expression in males during gonadogenesis in mice [39] which might be in accordance with the results in this study where we see low expression (close to the detection level) in the low expresser group. In mice, *fig α *has been suggested to play a key role in preserving oocytes and normal ovarian development [39] and in medaka *fig α *is an oocyte specific marker [38].

In this study, the pattern of *ar *expression in zebrafish during sex determination and differentiation clearly segregated the fish into two groups. This is in accordance with a study in sea bass where differences in *ar *expression were first encountered at 150 dph and became especially marked at 250 dph (corresponding with the time of sex differentiation) with higher expression in a male dominant group compared to a female dominant group (male and female dominant groups were based on consecutive size grading) [22]. In the current study, we find peaks in ar expression in the high expresser group at 16 and 25 dph which is shortly after a peak in expression of *dmrt1 *is observed and shortly before a peak in *sox9a *expression. These peaks in expression of the different genes around 10–18 dph coincide with the expected initiation of transformation from ovary to testis in individuals developing into males. The difference in expression profiles between the two groups at the time of sex differentiation is most likely sex-related and corresponds to differences between males and females, suggesting an important role for *ar *in sex differentiation. In a recent study of zebrafish gonad development, Hossain et al (2007) found *ar *mRNA levels at 4 weeks post fertilization (their first measurement) to be similar in males and females. However, from 5–7 wpf the *ar *expression was higher in the transforming testis [24]. Furthermore, expression levels of *ar *mRNA at different developmental stages has been studied in hermaphrodite fish species such as the protogynous wrasse, *Halichoeres trimaculatus *[40] and the protandrous black porgy, *Acanthopagrus schlegeli *[41]. In the black porgy, both the testicular tissue of the bisexual gonad and the functional testis exhibited higher *ar *mRNA levels than the ovarian tissue. Conversely, *ar *mRNA levels were lower in functional ovaries than in the ovarian part of the bisexual gonad, suggesting association of the decrease in *ar *mRNA levels with protandrous sex change in this fish species [41]. The ubiquitous expression of *ar *in adult zebrafish of both sexes found in this study is in accordance with [24] and we found *ar *expression in all investigated tissue of adult zebrafish (gonad, brain, liver, kidney, skin, muscle, eye) of both males and females.

Also, the ovarian form of aromatase, *cyp19a1a *was suggested to be involved in sex determination and differentiation [33]. *cyp19a1a *expression was found in undifferentiated gonads at 17 dpf which is expected based on the juvenile ovarian gonads. However, at 31 dpf the differentiated ovary showed specific expression of *cyp19a1a *in cells surrounding the oocytes [33]. Furthermore, at 31 dpf the differentiated testis showed no expression of *cyp19a1a *[33]. The results presented in this study correspond well with the previous studies of *cyp19a1a *expression. We find two peaks in expression during the sex determination and differentiation period (at 18 and 30 dph) in the high expresser group. Furthermore, we find that the expression of *cyp19a1a *in both the high and low expresser group is very low (almost undetectable in the low expresser group) at the time of gonadal transformation at approximately 19–21 dph. In accordance, a recent study showed *cyp19a1a *expression around oocytes in juvenile ovary, this expression was down-regulated and could no longer be detected when gonadal transformation was initiated [42].

The inter-individual grouping of the expression of *cyp19a1b *during sex determination and differentiation into high and low expressers observed in the present study is in accordance with previous studies [1,27,43]. In the present study we see one distinct peak in *cyp19a1b *expression at 14 dph for the high expresser group. This does not correspond completely with the peaks found in the high expresser group during sex determination and differentiation in the study by Trant et al. (2001), where *cyp19a1b *peaks were seen at 4–5 dpf, 13 dpf (small peak) and 25 dpf and from 34 – 40 dpf increase (with 2.5 dpf = 0 dph) [1]. The small peak at 13 dpf is most likely corresponding to the peak we see at 14 dph. However, the general expression pattern is not similar in the two studies. The finding that individual *cyp19a1b *expression during sex determination and differentiation distributes into two groups indicates that the expression of *cyp19a1b *(the brain form) might be associated with sexual differentiation in zebrafish as previously suggested by Trant et al. (2001). In contrast, Gato-Kazeto et al. (2004) investigated high and low expressers of *cyp19a2 *(the brain form) and found that it did not correlate with sex in adult zebrafish [27] Furthermore, a recent study on *cyp19a1b *expression in brain of adult zebrafish found that the expression levels were in the same range in males and females [44]. This is in accordance with the present study that indicates high brain expression of *cyp19a1b *in adults of both sexes. Due to the conflicting results regarding the involvement of *cyp19a1b *in gonadal sex differentiation, *cyp19a1b *is not included in the calculation of ratios between expected male and female genes in this study.

The coinciding low expression of *sox9a*, *dmrt1*, *ar*, *fig α *and *cyp19a1a *(in the high expresser groups) at 19–20 dph is just prior to the time of oocyte apoptosis in individuals developing into males. This could indicate that genes involved in the sex differentiation might be down-regulated at this time in development. Interestingly, it has previously been shown that the *vas *gene (which is a germ cell marker) was also down-regulated at 23 dpf [45]. Furthermore, the genes expected to be highly expressed in females (*fig α *and *cyp19a1a*) both had increased expression levels from 22 dph. Also, two of the three genes expected to be highly expressed in males (*ar *and *sox9a*) have a peak in expression at 22 dph. The last gene expected to be highly expressed in males (*dmrt1*) does not have a significant peak in expression after the general down regulation at 19–20 dph; this could indicate that dmrt1 (which shows two significant peaks in expression before 19–20 dph, at 10 and 14 dph) is involved in gonadal sex differentiation upstream in the signalling cascade.

## Conclusion

In conclusion, the current study investigated the expression pattern of six genes previously suggested to be involved in sex differentiation in individual zebrafish during the period of sex determination and differentiation. The three genes expected to be highest expressed in males (*ar*, *sox9a *and *dmrt1*) segregated into two groups of high and low expressers, respectively. These groups are putatively based on difference in genetic sex and might indicate a possible sex-related difference in expression profiles by the time of sex determination and gonadal differentiation. Thereby suggesting an important role for *ar*, *sox9a *and *dmrt1 *in controlling the male development process in zebrafish. Accordingly, the expression of the two genes expected to be highest expressed in females (*fig α *and *cyp19a1a*) also segregated fish into high and low expressers. Since the peaks in expression of these two genes are relatively late in the investigated period they are most likely involved in the gonadal sex differentiation of females and not in the sex determining process. The peak in expression of *dmrt1*, at 10 dph, suggests that *dmrt1 *might be upstream in the sex determining cascade, playing an important role in early gonadal sex differentiation of zebrafish males.

## Competing interests

The authors declare that they have no competing interests.

## Authors' contributions

AJ conducted the RNA purification, quantitative qRT-PCR and statistical analysis. JEM setup the zebrafish and collected the fish. OA, LJR and PB devised the study, participated in the discussion of results and participated in the planning of experiments. AJ wrote the manuscript. All authors read and approved the final manuscript.
